# Cuproptosis related genes associated with Jab1 shapes tumor microenvironment and pharmacological profile in nasopharyngeal carcinoma

**DOI:** 10.3389/fimmu.2022.989286

**Published:** 2022-12-23

**Authors:** Liping Wang, Dujuan Wang, Liu Yang, Xiaojiao Zeng, Qian Zhang, Guohong Liu, Yunbao Pan

**Affiliations:** ^1^ Department of Laboratory Medicine, Zhongnan Hospital of Wuhan University, Wuhan University, Wuhan, Hubei, China; ^2^ Department of Clinical Pathology, Houjie Hospital of Dongguan, the Affiliated Houjie Hospital of Guangdong Medical University, Dongguan, China; ^3^ Department of Radiology, Zhongnan Hospital of Wuhan University, Wuhan University, Wuhan, China

**Keywords:** copper death, tumor microenvironment, nasopharyngeal carcinoma, Jab1, immune infiltration

## Abstract

**Background:**

Nasopharyngeal carcinoma (NPC) is the most common subcategory of head and neck squamous cell carcinoma (HNSCC). This study focused on the roles of cuproptosis related genes and Jab1 in the tumor microenvironment of NPC and HNSCC.

**Methods:**

Differential expression analysis of Jab1 and cuproptosis related genes in tumor cell enriched region (PanCK-expressing) and immune cell enriched region (CD45-expressing) of NPC microenvironment were performed by packages of R software. Survival analysis was performed using the survival and survminer packages. Corrplot package was used for correlation analysis. ConsensusClusterPlus package was used for cluster clustering among different regions of NPC, and functional enrichment analysis was performed using GSVA, GSEABase, clusterProfiler, org.Hs.eg.db and enrichplot packages. The pRRophetic package was used to predict drug sensitivity in NPC and HNSCC.

**Results:**

Relationships exist between cuproptosis related genes and Jab1 in the NPC microenvironment. The expression of cuproptosis related genes and Jab1 differed between tumor cell enriched region and immune cell enriched region. AKT inhibitor VIII, Doxorubicin, Bleomycin and Etoposide showed higher sensitivity to tumor cell than immune cell. In the high Jab1 group, higher expression of ATP7A, DBT, DLD and LIAS were associated with better prognosis of HNSCC patients. In contrast, in the low Jab1 group, higher expression of these genes is associated with worse prognosis of HNSCC patients.

**Conclusions:**

Prognostic cuproptosis related genes and Jab1 provided a basis for targeted therapy and drug development.

## Introduction

The occurrence and development of a tumor depends not only on tumor cells themselves, but also on the soil in which the tumor cells live; i.e., the tumor microenvironment. The tumor microenvironment comprising tumor cells and tumor stroma (immune cells, fibroblasts, and endothelial cells) is the main battlefield for tumor occurrence and development, in which immune cells are important microenvironment components of tumor stroma (1). The NPC microenvironment is characterized by intense filtration of tumor-infiltrating immune cells (TIICs), which account for 40% to 50% of NPC tumor mass, with EBV-negative CD3+T lymphocytes being the most common infiltrators (2). Despite their abundance, an effective immune response is lacking and immunosuppressive infiltrates such as regulatory T cells (Tregs), M2 macrophages, and myeloid derived suppressor cells (MDSCs) are present, leading to immune tolerance and promoting tumor progression (3). How immune cells affected the occurrence and development of a tumor has always been a challenging problem in the field of tumor immunology.

EBV-associated NPC is usually a state of immune dysfunction (4). Furthermore, the first line of treatment is chemotherapy and radiotherapy, which tend to be immunosuppressive. This transition from suppression to immune activation is considered a prognostic factor. Cytokine-induced killer cell (CIK) and chimeric antigen receptor T-cell immunotherapy (CAR-T) have been used in EBV-associated hematologic tumors and have been validated at the *in vitro* level in the same EBV-associated NPC (5). CAR-T has shown potent antitumor activity in melanoma, leukemia, and lymphoma and is seen as a promising strategy for EBV-associated NPC. The use of LMP2A peptide as a vaccine has also been reported to benefit clinical outcomes in NPC patients (6). Enhanced CD8+ T-cell responses were observed in 9 of 16 NPC patients inoculated with autologous dendritic cells pulsed with LMP2 peptide epitopes. In addition, two of the 16 patients achieved a partial response (7). Combinations of immunotherapy and existing therapies need to be more personalized in the future and selected according to individual circumstances.

Copper is one of the essential trace elements in the human body and a cofactor of many essential enzymes in the organism. It has strong redox activity and protein binding ability, and it participates in the regulation of cellular physiological functions through the maintenance and regulation of intracellular copper homeostasis. Exposure to environmental exogenous factors can lead to a disorder of intracellular copper metabolism homeostasis, mediating cytotoxicity and body injury effects (8). It is known that the regulation of programmed cell death (PCD) or regulatory cell death (RCD) is the key to determining cell fate. The mechanisms of cytotoxicity and cell death induced by excessive copper exposure have not been fully elucidated. Recently, Tsvetkov et al. (9) confirmed that there exists copper-dependent, and regulated cell death in human cells, which is a new RCD mode that depends on mitochondrial respiration but is different from known cell death mechanisms. It occurs through direct binding of copper ions to lipoacylated components of the tricarboxylic acid cycle in mitochondrial respiration, resulting in aggregation of lipoacylated proteins and subsequent downregulation of iron-sulfur cluster proteins, resulting in proteotoxic stress and ultimately cell death. Several genes involved in copper-induced cell death were identified (10, 11), which may offer novel strategies to predict the prognosis of NPC patients.

It was reported that copper contents are closely related to NPC, and serum copper and ceruloplasmin levels may be used as markers for detection of NPC (12, 13). Additionally, recent research suggested that disulfiram/copper induce cytotoxicity and apoptosis in NPC cells and xenografts, which was highly probable to be mediated through ROS/MAPK pathways, and that the ROS scavenger N-acetyl-l-cysteine (NAC) could reverse the cellular and lipid ROS levels (14). The study by Ahmad et al. (15) demonstrated that Copper(II) ternary complex significantly inhibited tumor growth in nasopharyngeal carcinoma xenograft bearing mice models (15). The abovementioned lines of evidence showed the role of copper in the pathogenesis of NPC, which indicate that cuproptosis might be closely associated with NPC as well, providing insight into discovering novel therapies for NPC. One molecular mechanism leading to NPC tumorigenesis involves the fifth component of the COP9 signalosome complex (Csn5, COPS5 or Jab1). Jab1 acts as a modulator of intracellular signaling and affects cellular proliferation, apoptosis, and DNA damage response by interacting with several key regulatory proteins and affecting these proteins’ subcellular localization, degradation, phosphorylation, and deneddylation (16). Jab1 has been reported to be associated with cuproptosis related genes. For example, Jab1 is required for both Toll-like receptor and reactive oxygen species–mediated deneddylation of Cul3, which is essential for Cul3/Keap1-mediated degradation of NFE2L2 (17). Introducing the Jab1 deletion into the CDKN2A null background led to a complete rescue of liver structure and function and to a complete reversal of the DNA repair-associated genetic program resulting from Jab1 inactivation (18). The interaction between NLRP3 and Jab1 was also observed in THP-1 cells (19). We previously confirmed that overexpression of Jab1/COPS5 in NPC is associated with increased resistance to cancer therapies and poorer survival (18). However, no studies have yet reported the association between Jab1 and cuproptosis related genes in NPC or HNSCC.

This study focused on the differential expression of cuproptosis related genes and Jab1 in tumor-infiltrating immune cells and tumor cells and its relationship with the prognosis of NPC. We also evaluated the response of chemotherapeutic drugs in the immune cell enriched region and tumor cell enriched region. In order to further explore the role of Jab1/COPS5, we detected the correlation between Jab1/COPS5 and cuproptosis related genes in NPC, and explored the correlation between Jab1/COPS5 and sensitivity to chemotherapeutic drugs. It will provide a new idea for the study of copper death and prognosis of NPC.

## Methods

### Data collection

We collected biopsies from 41 untreated NPC samples from the Affiliated Houjie Hospital of Guangdong Medical University. These tissues were used to perform NanoString GeoMx DSP RNA assays at CapitalBio Technology (Beijing, China). This study was approved by the Ethics Scientific Committee of the Affiliated Houjie Hospital of Guangdong Medical University. Transcriptomic, clinical, and simple nucleotide variation data for head and neck squamous cell carcinoma (HNSCC) were obtained from The Cancer Genome Atlas (TCGA) database. Another commercial NPC TAM (Shanghai Mingyi Biotechnology Co., Ltd) containing 126 cases of NPC patients were used for Immunofluorescence analysis. A total of 108 cores were involved in follow-up analysis after the removal of unqualified cores and para-carcinoma tissue cores, as well as extreme values (< 5th percentile; > 95th percentile).

### Digital spatial profiling data generation and analysis

NanoString GeoMx DSP RNA assays were performed at CapitalBio Technology (Beijing, China) using the standard protocol. Briefly, to distinguish between various morphologies, tissue slides were stained with protein antibodies (against PanCK and CD45) and nuclear stain SYTO13 (Nanostring) was used. Regions of interest (ROIs) were placed based on the selection and assessment by a pathologist and illuminated using UV light. DSP assay sequencing data were processed with the GeoMx NGS Pipeline (DND). NPC microenvironment was divided into tumor cell-enriched region (PanCK-expressing), immune cell-enriched region (CD45-expressing), and normal epithelial (Epi) region.

### Heterogeneity of cuproptosis related genes and Jab1 in different regions

We obtained cuproptosis related genes based on previous studies (9, 10, 20). To explore the differences in expression of cuproptosis related genes and Jab1 in tumor cell-enriched region (PanCK-expressing), immune cell-enriched region (CD45-expressing), and normal epithelial (Epi) region, we performed differential analysis by limma, reshape2, ggplot2, and ggpubr packages using the Kruskal-Wallis test. The association of expression of cuproptosis related genes with NPC patient survival in the tumor cell-enriched region and immune cell-enriched region was analyzed by survival and survminer packages with the log-rank test, respectively. Differential expression of Jab1/COPS5 in tumor cell-enriched region, immune cell-enriched region, and normal epithelial region was analyzed by limma, ggplot2, and ggpubr packages using the Wilcoxon test. Survival analysis was performed using the survival and survminer packages, and the log-rank test was used to analyze the association of Jab1 with the survival of NPC patients in tumor cell-enriched region and immune cell-enriched region. For survival analysis, cuproptosis related genes and Jab1 grouping were determined based on the cutoff values, which were obtained through surv_cutpoint functions. Limma, corrplot, ggpubr, and ggExtra packages were used to explore the correlation between cuproptosis related genes and Jab1 in different regions using the Spearman method.

### Cuproptosis related genes for typing in tumor cell-enriched region and immune cell-enriched region of NPC

Principal component analysis (PCA) of cuproptosis related genes expression in 40 tumor cell-enriched regions and 39 immune cell-enriched regions were performed by prcomp and predict functions, and a score was calculated for each patient. The surv_cutpoint function was used to obtain the cutoff value of the score and it was divided into two groups. Percentage plots of the number of NPC patients according to the clinical parameters were drawn between the high and low score groups using plyr, ggplot2, and ggpubr packages. Difference analysis was performed between the high and low score groups, and differential genes were filtered by univariate Cox analysis for clustering in tumor cell-enriched region and immune cell-enriched region. The Wilcoxon test was used for the differential analysis. The R packages involved in differential, univariate Cox, and clustering analysis were as follows: limma, pheatmap, survival, survminer, and ConsensusClusterPlus. Interfractional Gene Set Variation Analysis (GSVA) analysis of tumor cell-enriched region and immune cell-enriched region was performed using pheatmap, GSEABase, GSVA, and limma packages.

### Drug sensitivity analysis

The pRRophetic package can predict tumor chemotherapy response based on gene expression levels. The main principle of the package is to build statistical models based on gene expression and drug sensitivity data in large cancer cell lines and apply the models to tumor gene expression matrices (21). The pRRophetic package was used to predict drug sensitivity in NPC and HNSCC patients. The difference in half maximal inhibitory concentration (IC50) between tumor cell-enriched region and immune cell-enriched region in NPC patients was analyzed using the Wilcoxon test. Transcriptomic data of HNSCC patients were obtained from the TCGA database. A total of 504 HNSCC samples were divided into two groups (Jab1 High and Jab1 Low) based on the median value of Jab1 expression. Correlation analysis between Jab1 expression and IC50 was performed using the Spearman method. R packages involved in differential analysis and plotting were as follows: limma, ggpubr, and ggplot2.

### The role of Jab1 in different areas of NPC

The different regions of NPC were grouped separately according to the median value of Jab1 expression. Differential analysis was performed in tumor cell-enriched region and immune cell-enriched region separately for the high and low Jab1 groups using limma, pheatmap, and ggplot2 packages with Wilcoxon test (log FC = 1, *P* < 0.05). Then survival analysis was conducted for respective differential genes by limma, survival, and survminer packages using the log-rank test to screen for prognostic genes in tumor cell-enriched region (PanCK-expressing) and immune cell-enriched region. Gene Ontology (GO) and Kyoto Encyclopedia of Genes and Genomes (KEGG) enrichment analyses of differential genes in tumor cell-enriched region and immune cell-enriched region were performed by clusterProfiler, org.Hs.eg.db, enrichplot, and ggplot2 packages. Stratification by Jab1 expression and region (tumor cell-enriched region (PanCK-expressing) and immune cell-enriched region), correlation analysis of cuproptosis related genes, COPS family genes, and differential genes (Differential genes of Jab1 high and low groups in tumor cell-enriched region and immune cell-enriched region) were performed using corrplot, ggpubr, and ggExtra packages with Spearman method.

### Mutation analysis

The TMB data obtained from the TCGA database, the cutoff value of TMB is obtained and grouped by the surv_cutpoint function. We explore the association of TMB and the prognosis of HNSCC patients with survival and survminer packages. HNSCC samples were stratified according to Jab1 expression (median value) and TMB for survival analysis.

### Expression of cuproptosis related genes in HNSCC patients with different Jab1 levels

HNSCC samples were divided into two groups according to the median value of Jab1 expression, with one group showing high expression of Jab1 and the other group showing the opposite trend. Differential expression of cuproptosis related genes between the two groups was explored by limma, reshape2, ggplot2, and ggpubr packages with Wilcoxon test. Subsequently, survival analysis of cuproptosis related genes in 252 HNSCC samples (Jab1 high) and 252 HNSCC samples (Jab1 low) was performed using the survival and survminer packages with the log-rank test. Groups in the survival analysis were determined based on the cutoff value, which was obtained through the surv_cutpoint functions.

### Multi-platform data validation

At the RNA level, we obtained 31 samples in NPC from the GEO database (GSE12452) to verify the relationship between cuproptosis related genes and Jab1. At the protein level, we validated the relationship between cuproptosis related genes and Jab1 in NPC by multiplex immunofluorescence and the prognostic impact of cuproptosis related genes and Jab1 on NPC patients.

## Results

### Heterogeneity of cuproptosis related genes and Jab1 in the NPC microenvironment

**Figure 1:**
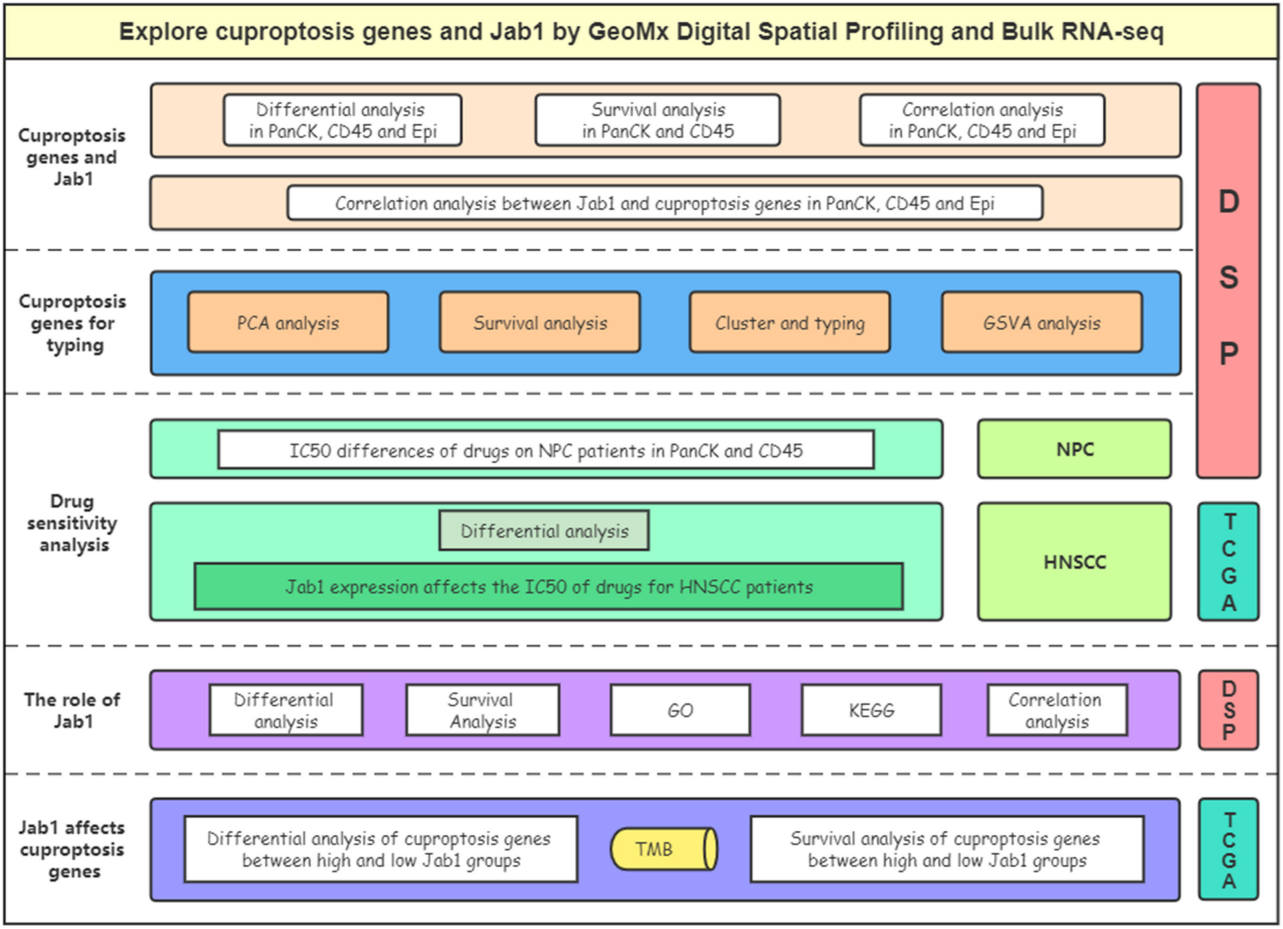
Flow chart of the study.

**Figure 2:**
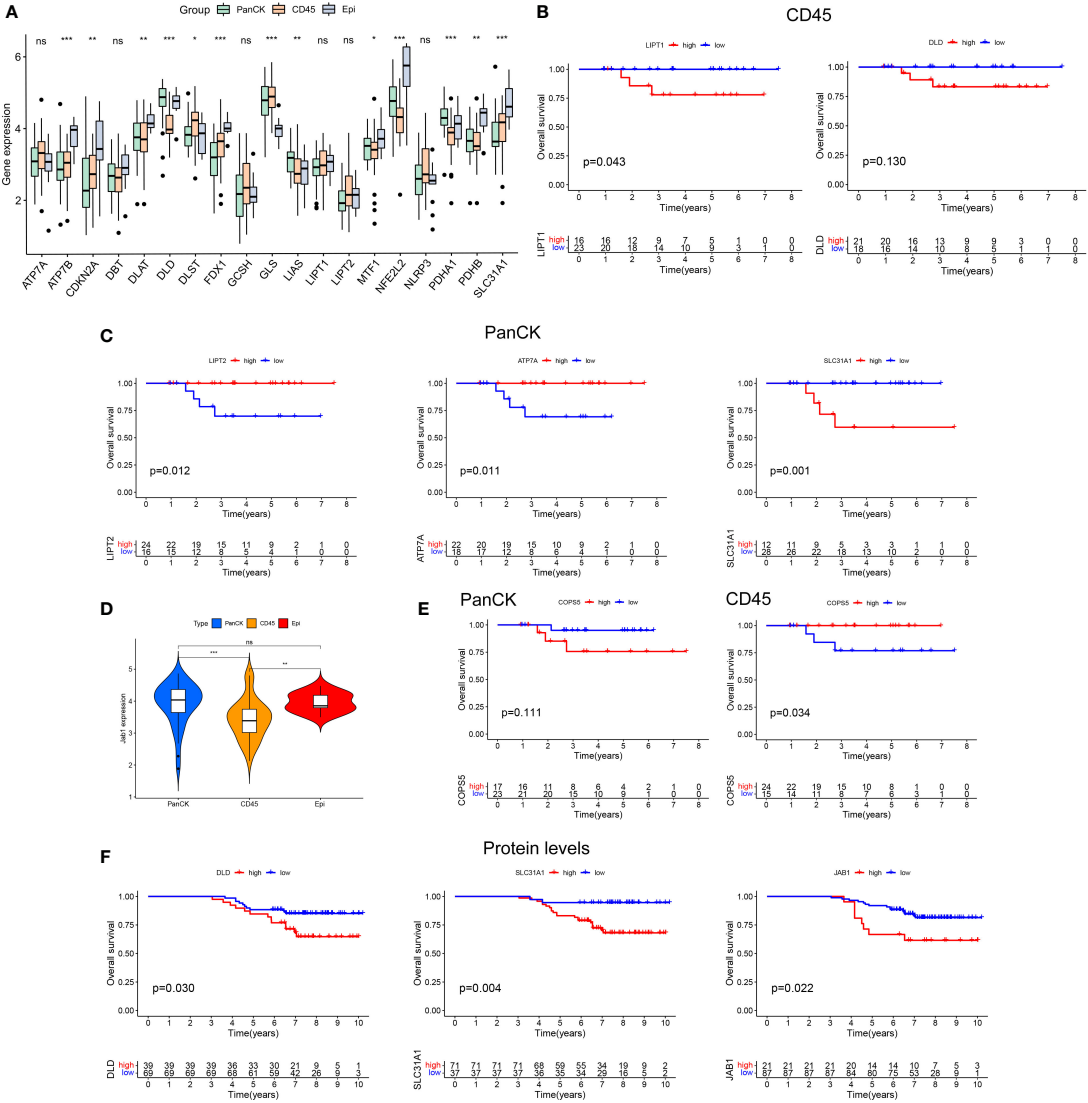
Heterogeneity of cuproptosis related genes and Jab1 in different regions of NPC tumor microenviroment. **(A)** Differential expression of cuproptosis related genes in three regions (tumor cell-enriched region, immune cell-enriched region, and normal epithelial region). **(B)** Cuproptosis related genes affect the survival of NPC patients in immune cell-enriched region. **(C)** Cuproptosis related genes affect the survival of NPC patients in tumor cell-enriched region. **(D)** Differential expression of Jab1 in three regions (tumor cell-enriched region, immune cell-enriched region, and normal epithelial region). **(E)** Jab1 affects the survival of NPC patients in tumor cell-enriched region and immune cell-enriched region. **(F)** Higher protein expression levels of cuproptosis related genes and Jab1 affect the survival of NPC patients. *p<0.05, **p<0.01, ***p<0.001, ns, not significant.

The procedure of this study is presented in **Figure 1**. The expression levels of ATP7B, CDKN2A, DLAT, DLD, DLST, FDX1, GLS, LIAS, MTF1, NFE2L2, PDHA1, PDHB, and SLC31A1 differed in the three regions with the Kruskal-Wallis test (*P* < 0.05). ATP7B, CDKN2A, FDX1, and SLC31A1 had the lowest expression in tumor cell-enriched region (PanCK-expressing). DLD, LIAS, and PDHA1 had the highest expression in tumor cell-enriched region (PanCK-expressing). DLAT, DLD, LIAS, MTF1, NFE2L2, PDHA1, and PDHB had the lowest expression in immune cell-enriched region. DLST and GLS had the highest expression in immune cell-enriched region (**Figure 2A**). NPC patients with higher LIPT1 (*P* = 0.043) and DLD (*P* = 0.130) expression in immune cell-enriched region had worse prognosis (**Figure 2B**). In tumor cell-enriched region, higher expression of SLC31A1 (*P* = 0.001) was associated with worse prognosis of NPC patients, while the opposite was true for LIPT2 (*P* = 0.012) and ATP7A (*P* = 0.011) (**Figure 2C**). Jab1 expression in tumor cell-enriched region was higher than that in immune cell-enriched region (*P* < 0.001). Jab1 expression in immune cell-enriched region is lower than that in normal epithelial region (*P* < 0.01) with the Wilcoxon test (**Figure 2D**). NPC patients with higher Jab1 expression in immune cell-enriched region had better prognosis (*P* = 0.034). In contrast, Jab1 played the opposite role in tumor cell-enriched region. Although the statistical significance of Jab1 in tumor cell-enriched region affecting the survival of NPC patients was not remarkable, it showed that Jab1 plays different roles in different locations of the NPC microenvironment (**Figure 2E**). At the protein level, higher levels of DLD, SLC31A1 and JAB1 are associated with worse prognosis for NPC patients (**Figure 2F**).

### The relationship between cuproptosis related genes and Jab1 in NPC

**Figure 3:**
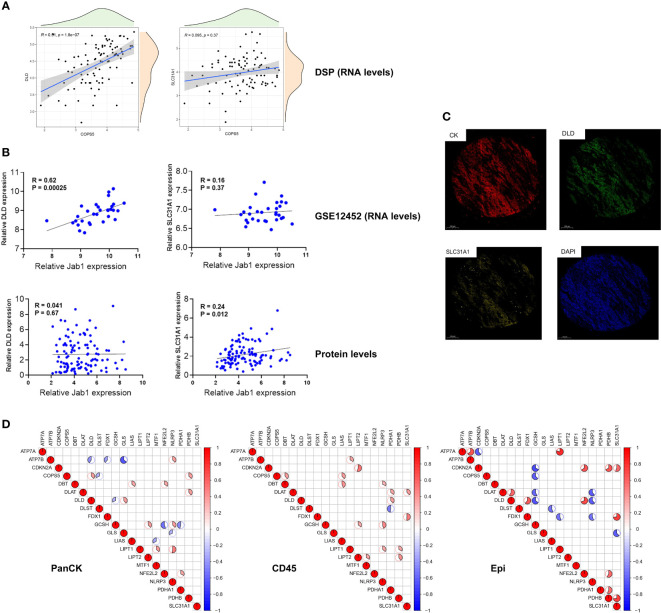
The relationship between cuproptosis related genes and Jab1 in NPC Correlation analysis of cuproptosis related genes and Jab1/COPS5 expressions in RNA **(A, B)** and protein levels **(B, C)**. **(D)** Correlation analysis of cuproptosis related genes and Jab1/COPS5 expressions in three regions (tumor cell-enriched region, immune cell-enriched region, and normal epithelial region). Red (positive correlation), blue (negative correlation), sector direction (clockwise for positive correlation, and counterclockwise for negative correlation), only the sector area at p < 0.05 is shown. The darker the colors, the larger the sector areas, and the higher the correlations.

**Figure 4:**
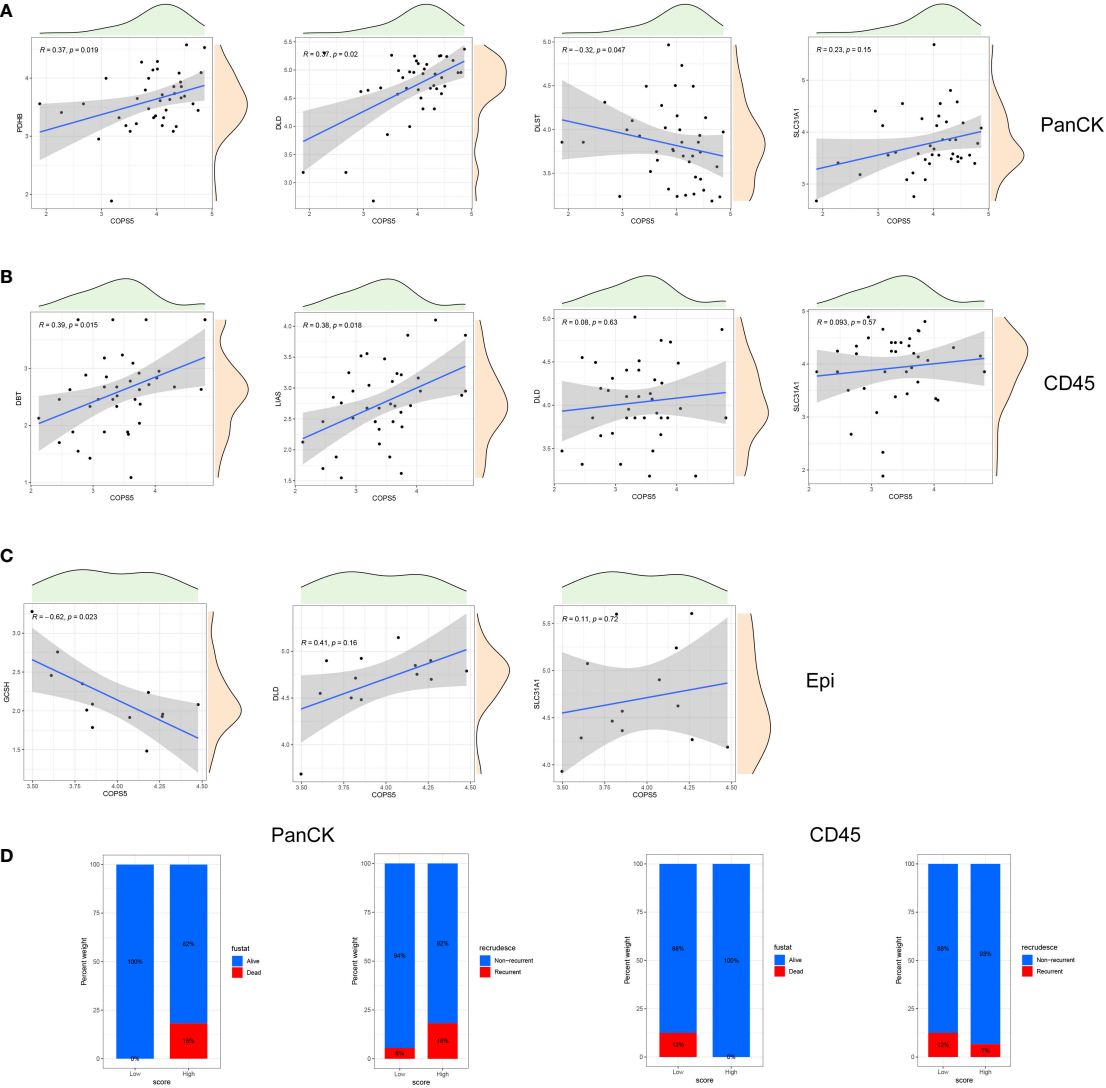
Correlation of cuproptosis related genes and Jab1 in different regions. **(A)** A correlation scatter plot: Jab1 correlates with the expression of cuproptosis related genes in tumor cell-enriched region. **(B)** A correlation scatter plot: Jab1 correlates with the expression of cuproptosis related genes in immune cell-enriched region. **(C)** A correlation scatter plot: Jab1 correlates with the expression of cuproptosis related genes in normal epithelial region. **(D)** NPC patient status distribution (PCA group) in tumor cell-enriched region and immune cell-enriched region.

Both at the RNA and protein levels, cuproptosis related genes (DLD and SLC31A1) are positively correlated with Jab1 as verified mutually by the three datasets (**Figures 3A, B**). As shown in **Figures 3C, D**, cuproptosis related genes and Jab1/COPS5 had different associations in three regions. Jab1 expression is positively correlated with PDHB (*R* = 0.37, *P* = 0.019), DLD (*R* = 0.37, *P* = 0.02) and SLC31A1 (*R* = 0.23, *P* = 0.15) expression while negatively correlates with DLST (*R* = -0.32, *P* = 0.047) expression in tumor cell-enriched region (**Figure 4A**). In immune cell-enriched region, DBT (*R* = 0.39, *P* = 0.015), LIAS (*R* = 0.38, *P* = 0.018), DLD (*R* = 0.08, *P* = 0.63) and SLC31A1 (*R* = 0.093, *P* = 0.57) expression are positively correlated with Jab1 expression (**Figure 4B**). In normal epithelial region, Jab1 expression is negatively correlated with GCSH (*R* = -0.62, *P* = 0.023) expression. Jab1 expression is positively correlated with DLD (R = 0.41, P = 0.16) and SLC31A1 (R = 0.11, P = 0.72) expression (**Figure 4C**).

### Cuproptosis related genes for stratification in the NPC microenvironment

**Figure 5:**
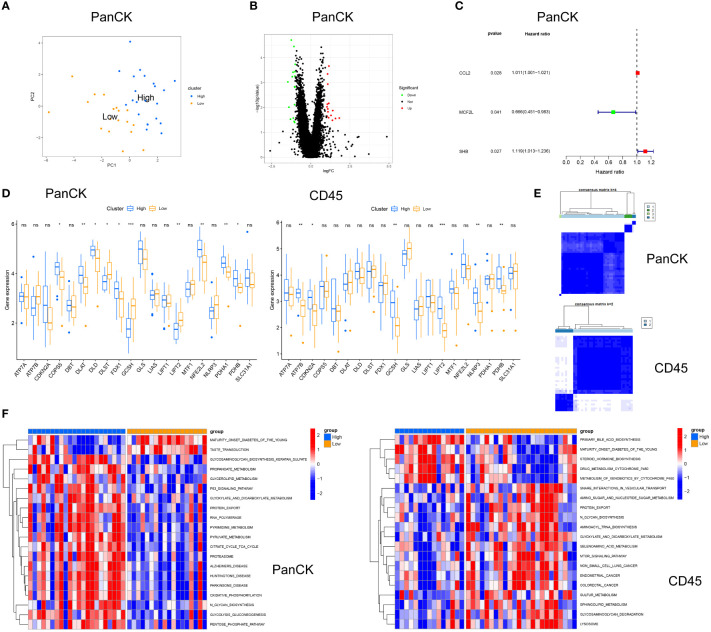
Cuproptosis related genes for stratification in tumor cell-enriched region and immune cell-enriched regions. **(A)** Principal Component Analysis in tumor cell-enriched region. **(B)** Differential analysis in Tumor cell-enriched region, green for downregulation, red for upregulation, log|FC|>1 and P<0.05. **(C)** Univariate Cox analysis in tumor cell-enriched region. **(D)** Differential expression of cuproptosis related genes and Jab1 between high and low score groups. **(E)** Typing in Tumor cell-enriched region: four clusters. Typing in immune cell-enriched region: two clusters **(F)** GSVA for high and low score groups in tumor cell-enriched regions and immune cell-enriched regions. *p<0.05, **p<0.01, ***p<0.001, ns, not significant.

PCA analysis was performed in 40 tumor cell-enriched regions and 39 immune cell-enriched regions according to the expression of cuproptosis related genes (**Figures 5A**, **S1D**). The clinical trait distribution of NPC patients in tumor cell-enriched region and immune cell-enriched region after PCA grouping is shown in **Figure 4D** and **Supplementary Figure 1A**. The scores derived from the PCA analysis are used to get the cutoff values and grouped by the surv_cutpoint function. We also explored the expression of cuproptosis related genes and Jab1 in different regions and PCA groupings. As shown in **Figure 5D**, in tumor cell-enriched region, the expression of COPS5, DLAT, DLD, FDX1, NFE2L2, PDHA1 and PDHB in the high score group is higher than that in the low score group, while the opposite is true for DLST, GCSH and LIPT2 (*P <*0.05). In immune cell-enriched region, the expression of ATP7B, CDKN2A, GCSH, LIPT2, NLRP3 and PDHB is higher in the high score group than in the low score group (*P <*0.05). By GSVA analysis, in tumor cell-enriched region, the high score group is mainly enriched in P53 signaling pathway, oxidative phosphorylation and metabolic related pathways. In immune cell-enriched region, the low score group is mainly enriched in tumor and metabolism-related pathways (**Figure 5F**). Differential genes screened by differential analysis between subgroups (|logFC|> 1 and *P <*0.05) for univariate cox analysis (*P <*0.05) to screen three prognostic genes (*CCL2*, *MCF2L* and *SHB*) in tumor cell-enriched region and four prognostic genes (*FAM169B, KRT27, NT5M* and *TIMP1*) in immune cell-enriched region (**Figures 5B, C**, **S1B, S1C, S1E**). We clustered NPC patients according to these prognostic genes in tumor cell-enriched region and immune cell-enriched region separately by ConsensusClusterPlus package (**Figure 5E**). We found that there are different subtypes existing in different regions, and a large heterogeneity is observed in different regions of NPC tumors.

### Drug sensitivity analysis

**Figure 6:**
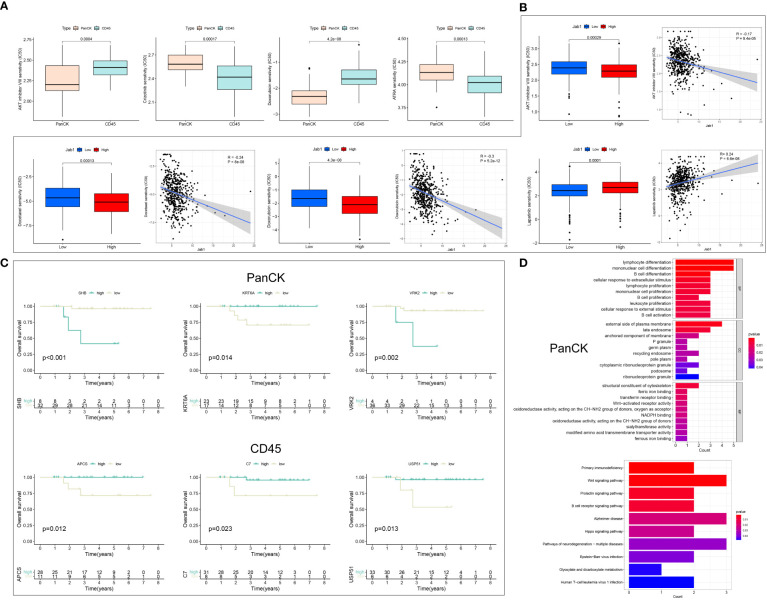
Drug sensitivity analysis and the role of Jab1 in different regions of NPC. **(A)** Differences in IC50 of drugs in NPC patients in tumor cell enriched regions and immune cell enriched regions. **(B)** Jab1 expression affects the IC50 of drug-treated HNSCC patients. **(C)** Differential genes from the Jab1 high and low expression groups for survival analysis in tumor cell enriched regions and immune cell enriched regions. **(D)** Differential genes from the Jab1 high and low expression groups for functional enrichment analysis (GO and KEGG) in tumor cell enriched regions.

In NPC patients, AKT inhibitor VIII, Doxorubicin, Bleomycin, and Etoposide showed higher sensitivity to tumor cell-enriched region than immune cell-enriched region (*P* < 0.001). Crizotinib, ATRA, Sunitinib, and Temsirolimus showed higher sensitivity to immune cell-enriched region (*P* < 0.001) than tumor cell-enriched region (**Figures 6A**, **S2A**). In HNSCC patients, the IC50 of Lapatinib was increased with high Jab1 expression (*P* < 0.001). In contrast, Jab1 expression was negatively correlated with the IC50 of AKT inhibitor VIII, Bleomycin, Docetaxel, Doxorubicin, Etoposide, Pyrimethamine, and Sorafenib (*P* < 0.001) (**Figures 6B**, **S2B**). The sensitivity of different regions of NPC patients to other drugs and the effect of Jab1 expression on the sensitivity of other drugs in HNSCC patients are presented in **Supplementary Tables 1**, **2**.

### The role of Jab1 in the NPC microenvironment

Differential analysis of the high and low Jab1 groups was performed in tumor cell enriched region and immune cell enriched region, respectively, and 25 (tumor cell-enriched region) and 23 (immune cell-enriched region) differential genes were obtained (**Figure S2C**). Survival analysis was performed for 25 (tumor cell-enriched region) and 23 (immune cell-enriched region) differential genes. In tumor cell enriched regions, SHB, VRK2, KRT6A, GRHPR, CGA, SLC6A8, and LY6D were associated with the survival of NPC patients (*P* < 0.05). In immune cell enriched regions, STAG3, APCS, USP51, C7, CACNB1, and GLS2 were associated with the survival of NPC patients (*P* < 0.05) (**Figures 6C**, **S2D**). Twenty-five (tumor cell-enriched region) differential genes were mainly involved in immune cell activation, proliferation, and differentiation. KEGG results showed that these 25 genes were mainly enriched in Epstein−Barr virus (EBV) infection, Wnt signaling pathway, and B cell receptor signaling pathway. It is suggested that Jab1 in tumor cell-enriched region was closely related to EBV and immune cell function (**Figure 6D**). Twenty-three (immune cell-enriched region) differential genes were mainly enriched in complement activation, humoral immunity, and amino acid and proteoglycan metabolism. In immune cell enriched regions, Jab1 expression could influence the complement, B-cells, and amino acid metabolism within this region (**Figure S2E**). In immune cell enriched regions, ARPP21 was positively correlated with ODAM expression when Jab1 was highly expressed, while ARPP21 was negatively correlated with ODAM expression when Jab1 was lowly expressed. C1orf94 was positively correlated with GLS2 expression when Jab1 was highly expressed, while C1orf94 was negatively correlated with GLS2 expression when Jab1 was lowly expressed.

**Figure 7:**
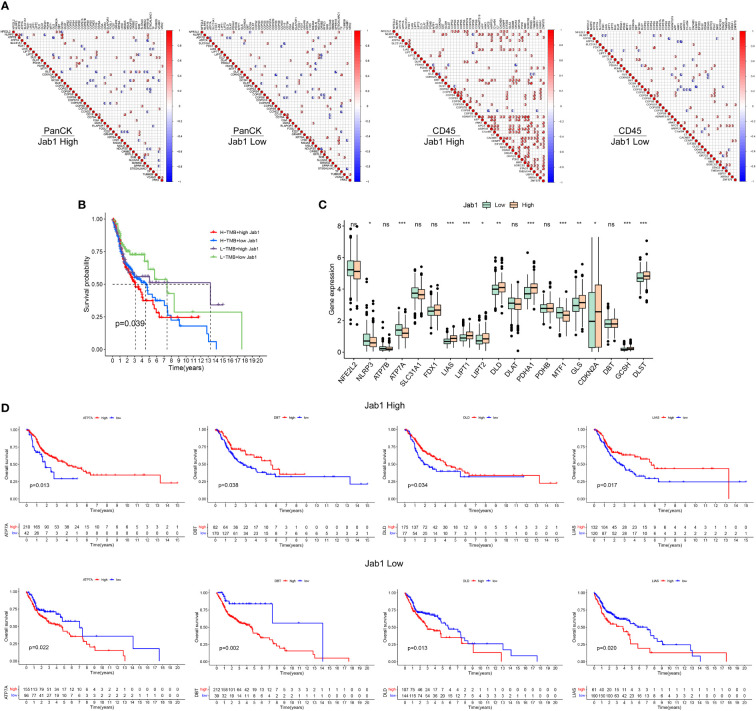
Differential analysis of cuproptosis related genes between the high and low Jab1 groups. **(A)** Correlation analysis of cuproptosis related genes, COPS family genes, and differential genes from the high and low Jab1 groups in different regions (tumor cell-enriched region and immune cell-enriched region) and different Jab1 subgroups: red (positive correlation), blue (negative correlation), sector direction (clockwise for positive correlation, and counterclockwise for negative correlation), only the sector area at p < 0.05 is shown. The darker the colors, the larger the sector areas, and the higher the correlations. **(B)** TMB and Jab1 affect survival of HNSCC patients. **(C)** Differential analysis of cuproptosis related genes between the high and low Jab1 groups. **(D)** Cuproptosis related genes and Jab1 affect the survival of HNSCC patients. *p<0.05, **p<0.01, ***p<0.001, ns, not significant.

With low expression of Jab1, NFE2L2 was negatively correlated with GCSH expression in tumor cell enriched regions, while NFE2L2 was positively correlated with GCSH expression in immune cell enriched regions. These results suggested that the expression patterns of genes differed between different regions in NPC and Jab1 may regulate these genes in different ways (**Figure 7A**).

### Jab1 associated with cuproptosis related genes in HNSCC patients

In the low TMB and low Jab1 groups, HNSCC patients showed the best prognosis, whereas in the high TMB and high Jab1 group, HNSCC patients showed the worst prognosis. When TMB was unchanged, higher Jab1 expression was associated with worse prognosis of HNSCC patients. When Jab1 expression was unchanged, greater TMB was associated with worse prognosis of HNSCC patients (*P* = 0.039, **Figure 7B**). In the low Jab1 group, the expression levels of NLRP3, ATP7A, and MTF1 were higher than those in the high Jab1 group (*P* < 0.05). In the high Jab1 group, the expression levels of LIAS, LIPT1, LIPT2, DLD, PDHA1, GLS, CDKN2A, and DLST were higher than those in the low Jab1 group (*P* < 0.05, **Figure 7C**). In the high Jab1 group, higher expression levels of ATP7A, DBT, DLD, and LIAS were associated with better prognosis of HNSCC patients. In contrast, in the low Jab1 group, higher expression levels of ATP7A, DBT, DLD, and LIAS were associated with worse prognosis of HNSCC patients (*P* < 0.05, **Figure 7D**). The prognostic impact of other cuproptosis related genes on HNSCC at different Jab1 expression levels can be seen in **Supplementary Figure S3**.

## Discussion

NPC is one of the common types of head and neck tumors with poor prognosis. Therefore, it is important to identify novel biomarkers that can predict the clinical outcome of NPC and to investigate the molecules involved in tumor progression for patient treatment.

Previous studies have shown that the expression level of Jab1 is elevated in many tumors, including NPC (16), which is consistent with the findings of our study. We found that Jab1 is elevated in the tumor cell-enriched regions compared with the normal epithelial-enriched regions. Interestingly, the present study found that Jab1 expression was lower in the immune cell-enriched regions. In the tumor microenvironment, Jab1 expression is not only different in different cell-enriched regions, but is also correlated with different cuproptosis related genes, suggesting that Jab1 plays different roles in the NPC tumor microenvironment. At both gene and protein levels, cuproptosis related genes (DLD and SLC31A1) show a positively correlated trend with Jab1. Higher DLD expression in immune cell infiltrated areas is associated with worse prognosis in NPC patients. Although not statistically significant, this may be related to the limited sample size. In tumor cell infiltrated areas, higher SLC31A1 expression leads to poorer prognosis of NPC. Increased protein expression of DLD, SLC31A1 and Jab1 tend to poorer prognosis. Our study found that Jab1 are associated with cuproptosis related genes in NPC.

Our study also showed that the tumor cell enrichment region and immune cell enrichment region have different reactivity to various drugs, and the Jab1 expression level is negatively correlated with the reactivity of many drugs. For example, the Jab1 expression level is negatively correlated with the reactivity of Doxorubicin, which is consistent with our previous report. Enrichment analysis showed that Jab1 differential expression groups were significantly enriched in immune cell differentiation, proliferation, and Wnt signaling pathway. Wnt is highly expressed in most tumor patients and can promote angiogenesis and tumor cell proliferation. Lymphocyte differentiation and proliferation play an important role in regulating drug resistance in the tumor microenvironment. In the NPC microenvironment, we found that Jab1 is mainly highly expressed in the tumor cell region and lowly expressed in the immune cell region. Functions performed by Jab1 in different regions are different. In the immune cell region, high Jab1 expression favors the prognosis of NPC patients, but in the tumor cell region, high Jab1 expression is detrimental to the survival of NPC patients. NPC is a type of HNSCC, and Jab1 is overexpressed in HNSCC, which promotes the proliferation and migration of HNSCC cells and is detrimental to the survival of HNSCC patients (22). We also found that Jab1 expression affects the efficacy of chemotherapy in HNSCC patients. Overexpression of Jab1 in mice can lead to spontaneous osteosarcoma formation in a p53-dependent manner (23).

Cuproptosis is a recently proposed new mode of cell death, in which copper binds directly to the lipidated components of the tricarboxylic acid (TCA) cycle leading to proteotoxic stress and ultimately cell death (8, 9, 24, 25). Previous studies have reported that cuproptosis related genes may serve as prognostic markers for predicting HNSCC (26). HNSCC resistance to platinum was associated with Copper-dependent ATP7B activity, while TMEM16A and ATP7B expression were positively correlated. The increased ATP7B in TMEM16A overexpressing cells could be reversed by inhibition of NADPH oxidase 2, antioxidant N-Acetyl-Cysteine and copper chelation. Increased oxidative stress in TMEM16A overexpressing cells releases chelated copper from the cytoplasm, leading to transcriptional activation of ATP7B expression. Overexpression of TMEM16A in HNSCC leads to upregulation of ATP7B and thus resistance to platinum drugs (27). Previous studies have also reported that the interaction between cuproptosis related genes and Jab1. The lack of COPS5 in regenerating livers triggers a CDKN2A-dependent genetic program leading to cell cycle arrest and apoptosis (18). Jab1 is involved in nuclear factor E2-related factor 2-mediated gene regulation and is required for Cul3/Keap1-mediated degradation of nuclear factor E2-related factor 2 (17). Thiolutin is an inhibitor of JAB1/MPN/Mov34 domain-containing metalloprotease and blocks NLRP3 inflammatory vesicle activation (28).

We speculate that perhaps the cuproptosis approach could be used to promote NPC or HNSCC cell death and increase the survival of patients. We found that the expression of these cuproptosis related genes differed in different regions of NPC. In the immune cell region, the patients with higher expression of LIPT1 had the worse prognosis. In the tumor cell region, the patients with higher expression of LIPT2 and ATP7A had the better prognosis; while the higher the expression of SLC31A1, the worse the prognosis of NPC patients. The association patterns of NFE2L2 and GCSH in the tumor cell region and immune cell region of NPC are different when Jab1 is lowly expressed. In the immune cell region of NPC, we also found that Jab1 influences the association of ARPP21-ODAM and C1orf94-GLS2. Jab1 influences the expression of cuproptosis related genes in HNSCC patients and affects patient prognosis. In the Jab1 highly expressed population, ATP7A, DBT, DLD, and LIAS expression levels are higher and the prognosis of HNSCC patients is better. However, in the Jab1 lowly expressed population, the results are completely opposite. The mechanism of cuproptosis in NPC and how Jab1 affects cuproptosis needs further study.

In conclusion, we explored the relationship between cuproptosis related genes and Jab1 and how they affect the prognosis of NPC and HNSCC patients. We also predicted the effect of chemotherapy in different regions of the NPC tumor microenvironment and assessed how Jab1 affects HNSCC chemotherapy. We investigated roles of cuproptosis related genes and Jab1 in NPC and HNSCC, and screened prognostic genes to provide a theoretical basis and possibility for treating patients with NPC and HNSCC in a cuproptosis manner.

## Data availability statement

The raw sequence data reported in this paper have been deposited in the Genome Sequence Archive in National Genomics Data Center, China National Center for Bioinformation / Beijing Institute of Genomics, Chinese Academy of Sciences (accession number: HRA003609) that are publicly accessible at https://ngdc.cncb.ac.cn/gsa-human/browse/HRA003609.

## Ethics statement

The studies involving human participants were reviewed and approved by Ethics Scientific Committee of the Affiliated Houjie Hospital of Guangdong Medical University. The patients/participants provided their written informed consent to participate in this study.

## Author contributions

LW: Methodology, Data curation, Writing—Original draft preparation. DW: Methodology and Data curation. LY: Validation. XZ: Validation. QZ: Validation. GL: Conceptualization, Data Curation, Writing—Reviewing, Editing and Funding acquisition. YP: Conceptualization, Supervision, Writing—Reviewing and Editing, Funding acquisition. All authors contributed to the article and approved the submitted version.

## Supplementary material

Supplementary Figure 1 Cuproptosis related genes for stratification in tumor cell-enriched region and immune cell-enriched region region. **(A)** NPC patient status distribution (PCA group) in tumor cell-enriched region and immune cell-enriched region. **(B)** Differential analysis in immune cell-enriched region, green for downregulation, red for upregulation, log|FC|>1 and P<0.05. **(C)** Univariate Cox analysis in immune cell-enriched region. **(D)** Principal component analysis in immune cell-enriched region. **(E)** Survival analysis of genes screened by univariate Cox regression analysis in tumor cell-enriched region and immune cell-enriched region.

Supplementary Figure 2 Drug sensitivity analysis and the role of Jab1 in different regions of NPC. **(A)** Differences in IC50 of drugs in NPC patients in tumor cell-enriched region and immune cell-enriched region. **(B)** Jab1 expression affects the IC50 of drug-treated HNSCC patients. **(C)** Differential analysis of the Jab1 high and low expression groups in tumor cell-enriched region and immune cell-enriched region. **(D)** Differential genes from the Jab1 high and low expression groups for survival analysis in tumor cell-enriched region and immune cell-enriched region. **(E)** Differential genes from the Jab1 high and low expression groups for functional enrichment analysis (GO and KEGG) in immune cell-enriched region.

Supplementary Figure 3 Effect of cuproptosis related genes on the survival of HNSCC patients with different Jab1 expression levels.
