# Supplementary material for: Cuproptosis related genes associated with Jab1 shapes tumor microenvironment and pharmacological profile in nasopharyngeal carcinoma
Source: Front Immunol. 2022 Dec 23;13:989286. doi: 10.3389/fimmu.2022.989286 (PMC9816571; doi:10.3389/fimmu.2022.989286)
Supplement: Supplementary file 1 [file Table_1.docx]

Supplementary table 1. Drug sensitivity in panCK and CD45 region

| Drugs | panCK | CD45 |
| --- | --- | --- |
| 681640 | * |  |
| A-443654 | * |  |
| AC220 |  | * |
| AKT inhibitor VIII | * |  |
| ATRA |  | * |
| AZD8055 |  | * |
| BEZ235 |  | * |
| BI-2536 | * |  |
| BIRB 0796 | * |  |
| Bleomycin (50 uM） | * |  |
| Bleomycin | * |  |
| BMS-754807 | * |  |
| Bosutinib | * |  |
| CAL-101 |  | * |
| Camptothecin | * |  |
| CCT007093 | * |  |
| Cetuximab | * |  |
| CGP-60474 |  | * |
| Cisplatin | * |  |
| CP724714 | * |  |
| Crizotinib |  | * |
| Cyclopamine |  | * |
| Cytarabine | * |  |
| DMOG |  | * |
| Docetaxel | * |  |
| Doxorubicin | * |  |
| EHT 1864 | * |  |
| Embelin | * |  |
| Epothilone B | * |  |
| Erlotinib | * |  |
| Etoposide | * |  |
| FH535 | * |  |
| Gefitinib | * |  |
| Gemcitabine | * |  |
| GSK-650394 | * |  |
| GW-2580 | * |  |
| GW 441756 | * |  |
| GW843682X | * |  |
| I-BET-762 |  | * |
| IOX2 | * |  |
| Ispinesib Mesylate | * |  |
| JNK Inhibitor VIII | * |  |
| JNK-9L | * |  |
| JQ12 | * |  |
| KIN001-055 | * |  |
| KIN001-102 |  | * |
| KIN001-260 |  | * |
| KIN001-270 |  | * |
| Mitomycin C | * |  |
| MK-2206 |  | * |
| MLN4924 | * |  |
| Nilotinib |  | * |
| NSC-87877 | * |  |
| Obatoclax Mesylate | * |  |
| OSU-03012 | * |  |
| Pazopanib |  | * |
| PF-562271 | * |  |
| Phenformin |  | * |
| PI-103 |  | * |
| piperlongumine |  | * |
| Pyrimethamine | * |  |
| QS11 | * |  |
| Rapamycin |  | * |
| RO-3306 | * |  |
| rTRAIL | * |  |
| SB 216763 | * |  |
| SB52334 | * |  |
| Shikonin | * |  |
| SN-38 | * |  |
| STF-62247 |  | * |
| Sunitinib |  | * |
| TAE684 |  | * |
| Temsirolimus |  | * |
| TG101348 |  | * |
| TGX221 |  | * |
| Thapsigargin | * |  |
| THZ-2-49 |  | * |
| Tipifarnib | * |  |
| TL-2-105 |  | * |
| TW 37 | * |  |
| Vinblastine | * |  |
| Vinorelbine | * |  |
| VX-680 | * |  |
| VX-702 |  | * |
| XAV939 | * |  |
| XMD8-85 |  | * |
| YK 4-279 | * |  |

*Lower IC50 value
