# Supplementary material for: Cuproptosis related genes associated with Jab1 shapes tumor microenvironment and pharmacological profile in nasopharyngeal carcinoma
Source: Front Immunol. 2022 Dec 23;13:989286. doi: 10.3389/fimmu.2022.989286 (PMC9816571; doi:10.3389/fimmu.2022.989286)
Supplement: Supplementary file 2 [file Table_2.docx]

Supplementary table 2. Correlation between Jab1 expression and drugs sensitivity

| Drugs | R | P |
| --- | --- | --- |
| 5−Fluorouracil | - 0.26 | 3.4e−09 |
| A-443654 | -0.22 | 7.4e-07 |
| A-770041 | 0.28 | 1.1e-07 |
| AKT inhibitor VIII | -0.17 | 9.4e-05 |
| ATRA | 0.22 | 5.2e-07 |
| AUY922 | -0.3 | 1e-11 |
| AZ628 | -0.28 | 1.5e-09 |
| BAY 61-3606 | -0.19 | 3e-05 |
| Bexarotene | -0.21 | 3.6e-06 |
| BI-2536 | -0.26 | 2.9e-09 |
| Bleomycin | -0.17 | 0.00011 |
| CCT018159 | -0.26 | 2.5e-09 |
| CX-5461 | -0.24 | 6.2e-08 |
| Dabrafenib | -0.25 | 1.8e-08 |
| Docetaxel | -0.24 | 8e-08 |
| Doxorubicin | -0.3 | 5.2e-12 |
| Elesclomol | -0.22 | 6e-07 |
| Epothilone B | -0.31 | 6.6e-13 |
| Etoposide | -0.33 | 8e-14 |
| EX-527 | -0.26 | 5.2e-09 |
| FH535 | -0.39 | ＜2.2e-16 |
| FK866 | -0.27 | 1.7e-09 |
| Gemcitabine | -0.24 | 9.4e-08 |
| GSK-650394 | -0.34 | 1.9e-15 |
| GSK1904529A | -0.23 | 1.3e-07 |
| GW843682X | -0.27 | 1.2e-09 |
| HG-6-64-1 | -0.22 | 7.6e-07 |
| Ispinesib Mesylate | -0.2 | 8.1e-06 |
| JNK-9L | -0.37 | ＜2.2e-16 |
| JQ12 | -0.31 | 1.5e-12 |
| KIN001-266 | -0.24 | 6.9e-08 |
| Lapatinib | 0.24 | 6.6e-08 |
| LAQ824 | -0.38 | ＜2.2e-16 |
| LFM-A13 | -0.17 | 0.00015 |
| Masitinib | -0.21 | 1.7e-06 |
| Mitomycin C | -0.24 | 9.7e-08 |
| MLN4924 | -0.23 | 1.9e-07 |
| MS-275 | -0.26 | 3.2e-09 |
| Obatoclax Mesylate | -0.32 | 4e-13 |
| OSI-027 | -0.21 | 2.6e-06 |
| OSU-03012 | -0.41 | ＜2.2e−16 |
| PAC-1 | -0.21 | 1.8e-06 |
| PF-562271 | -0.25 | 2.3e-08 |
| PHA-793887 | -0.2 | 5.6e-06 |
| Pyrimethamine | -0.32 | 1.7e-13 |
| QS11 | -0.31 | 6.1e-13 |
| Roscovitine | -0.21 | 2.8e-06 |
| Salubrinal | -0.3 | 8.1e-12 |
| Sorafenib | -0.26 | 2.9e-09 |
| TAK-715 | -0.27 | 1.6e-09 |
| Temozolomide | -0.2 | 4e-06 |
| Thapsigargin | -0.25 | 1.2e-08 |
| THZ-2-49 | 0.19 | 1.2e-05 |
| Tipifarnib | -0.34 | 3.8e-15 |
| Tubastatin A | -0.22 | 4.5e-07 |
| TW 37 | -0.26 | 5.7e-09 |
| Vinorelbine | -0.34 | 6.2e-15 |
| VX-680 | -0.23 | 1.1e-07 |
| XMD11-85h | -0.22 | 4.9e-07 |
| XMD13-2 | -0.19 | 1.7e-05 |
| YK 4-279 | -0.25 | 1.6e-08 |
| Z-LLNle-CHO | 0.24 | 6.4e-08 |
| ZM-447439 | -0.2 | 7.9e-06 |
