# Supplementary figures and images for: Cuproptosis related genes associated with Jab1 shapes tumor microenvironment and pharmacological profile in nasopharyngeal carcinoma
Source: Front Immunol. 2022 Dec 23;13:989286. doi: 10.3389/fimmu.2022.989286 (PMC9816571; doi:10.3389/fimmu.2022.989286)

Fig. s1

B

CD45

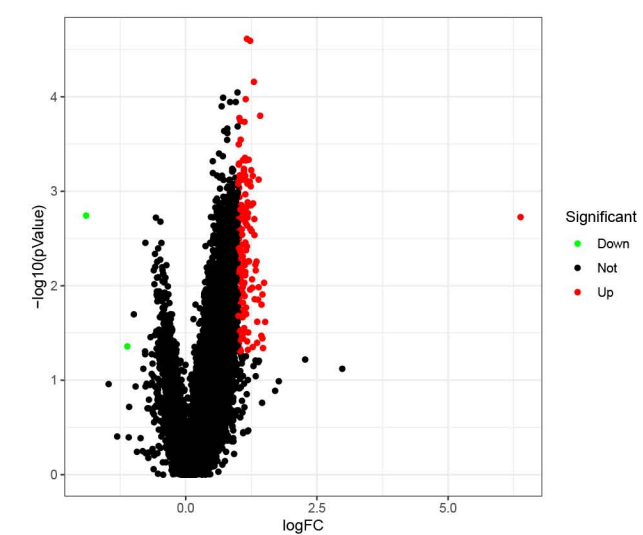

C

CD45

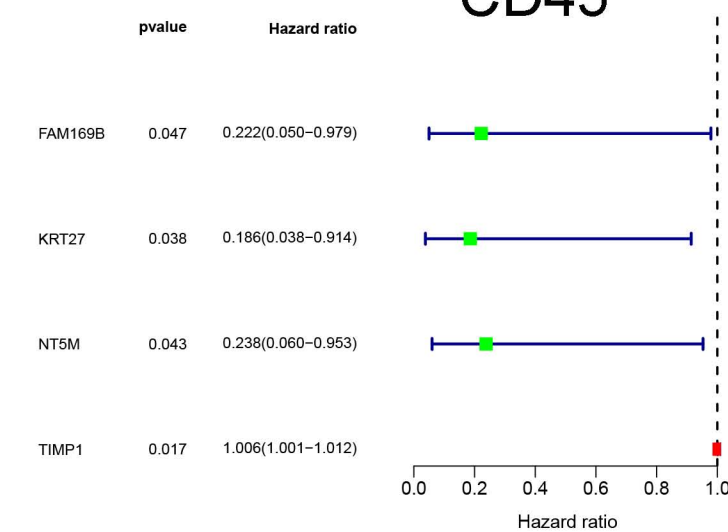

A

PanCK

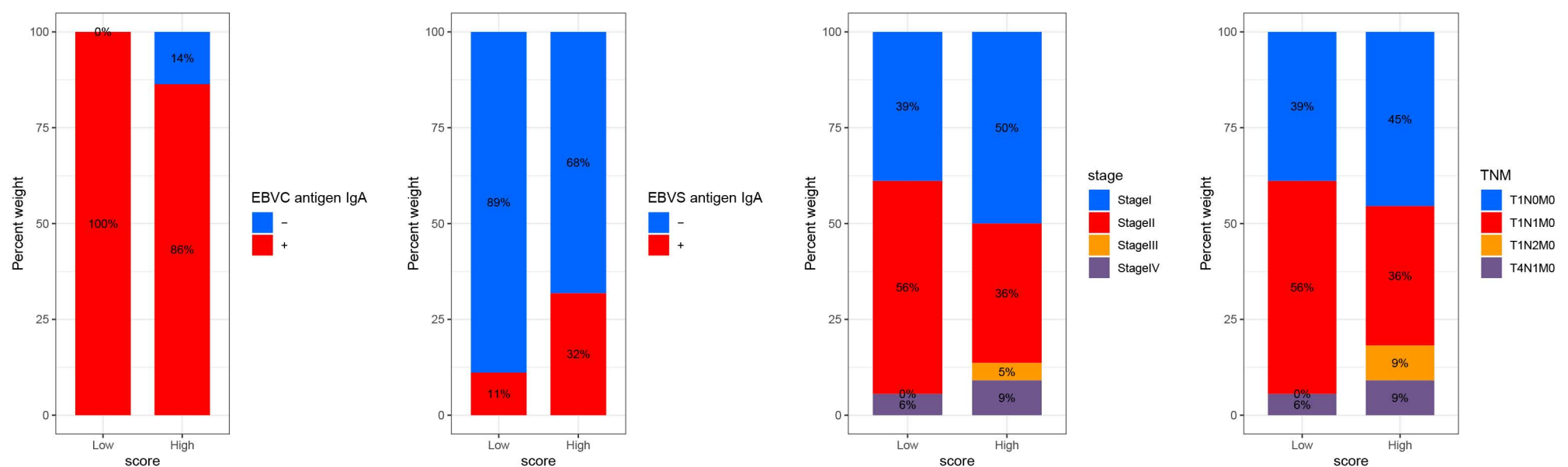

CD45

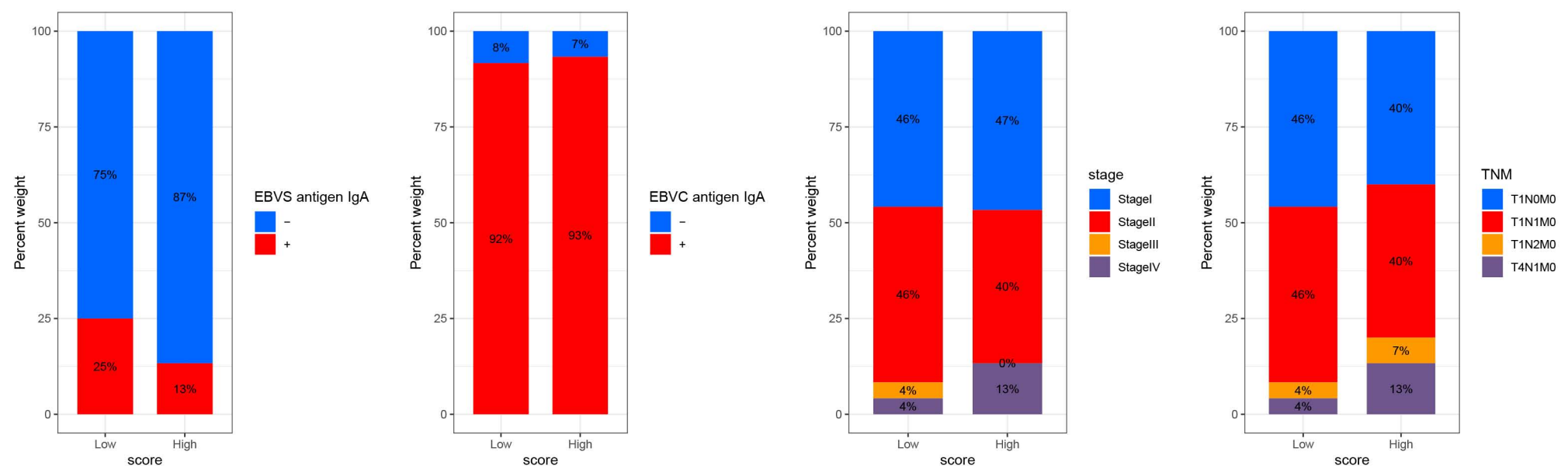

D

CD45

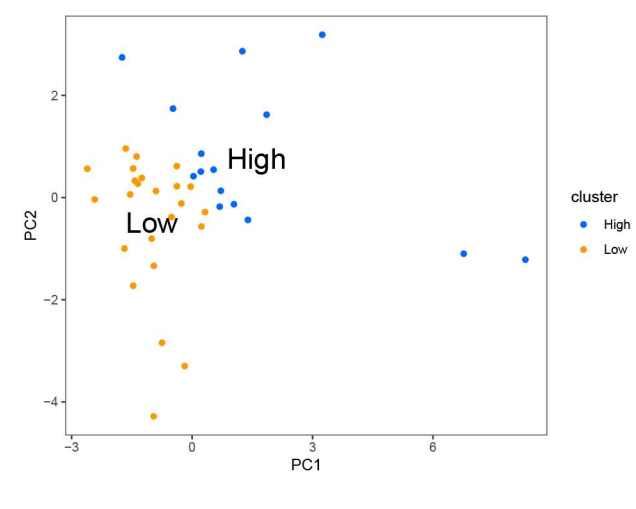

E

PanCK

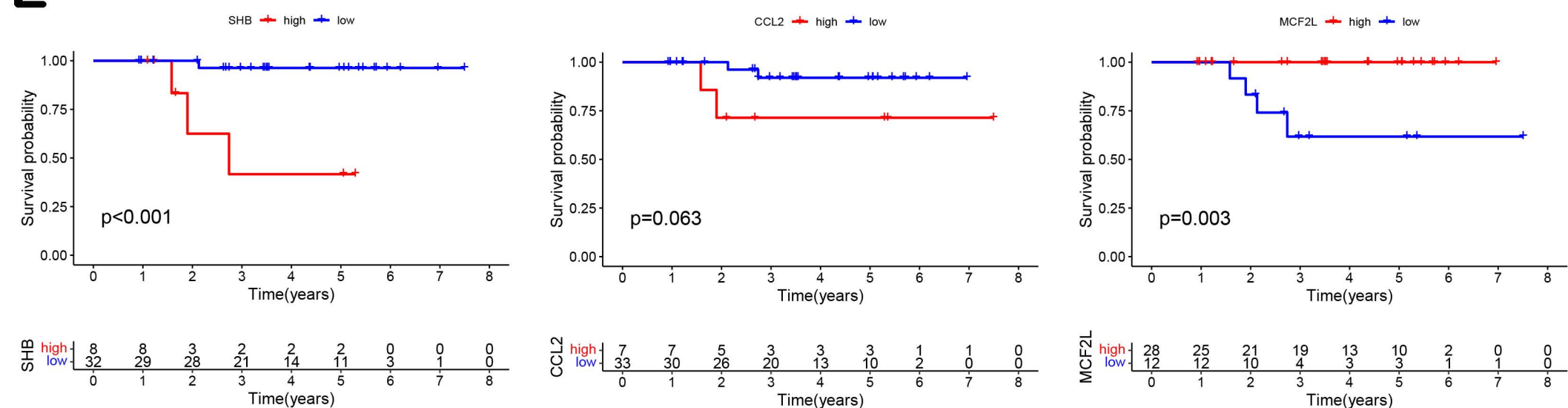

CD45

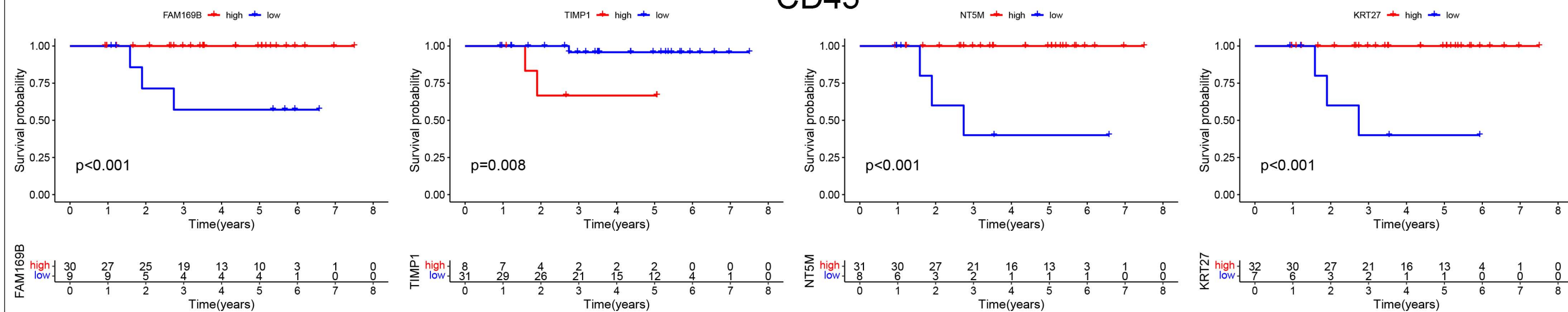

Fig. s2

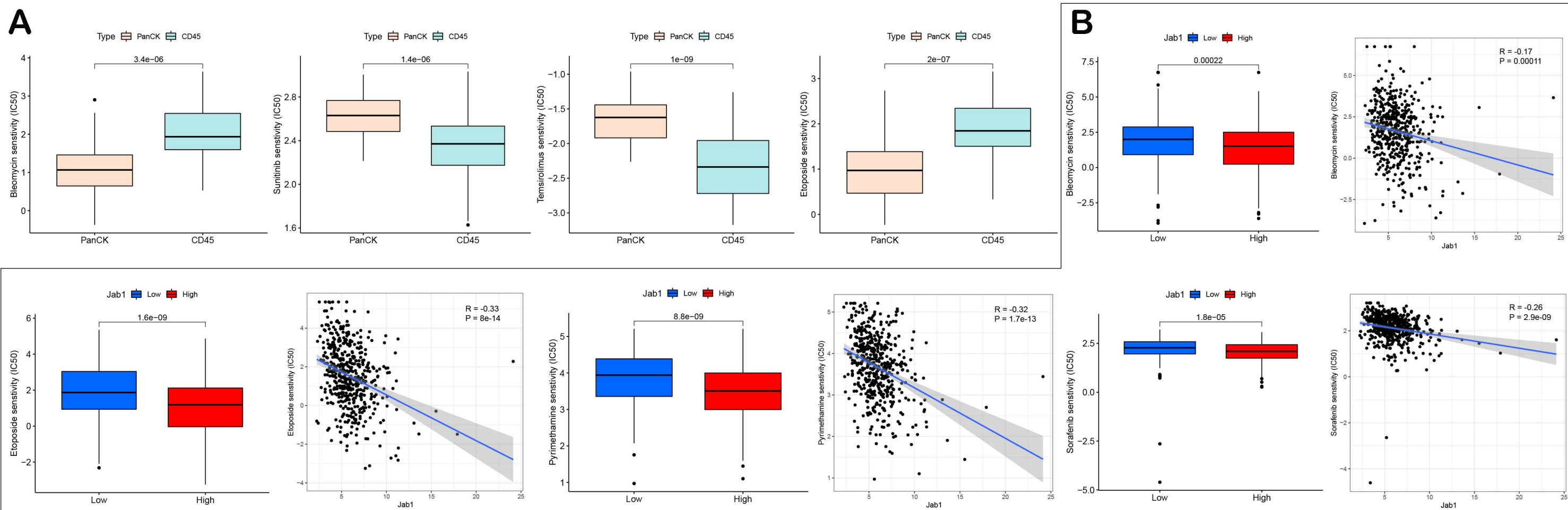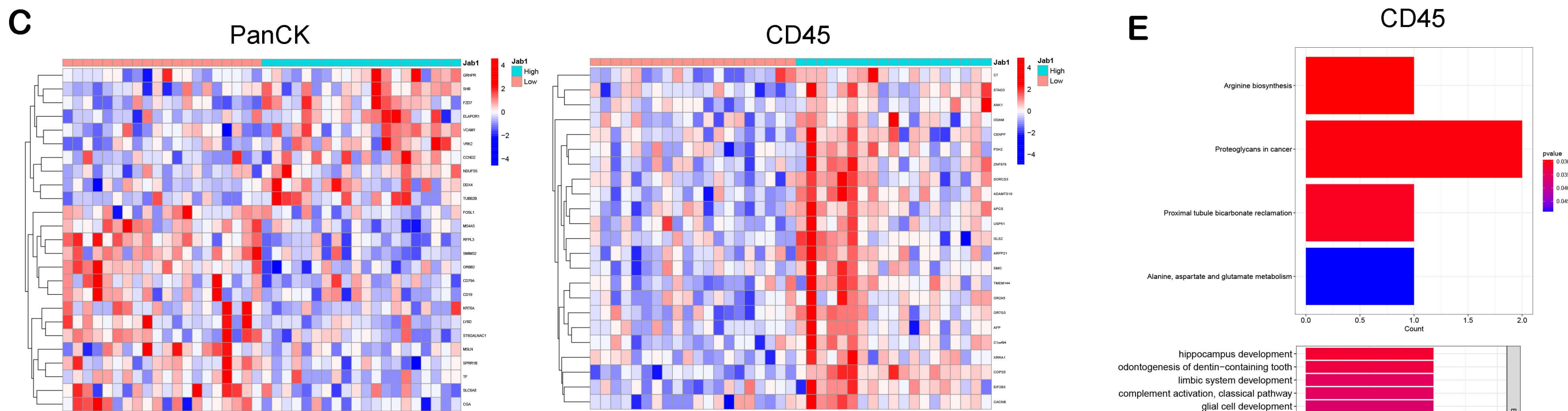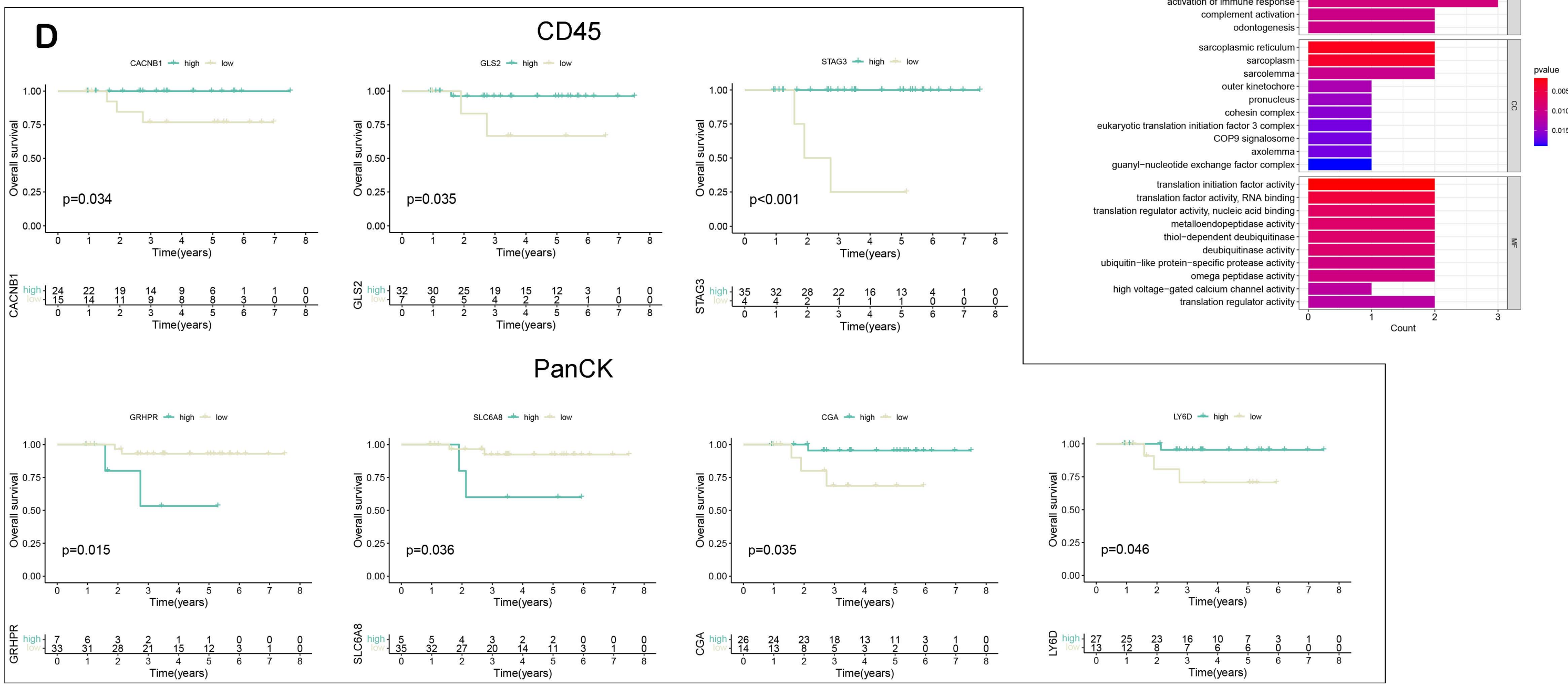

Jab1 High

A

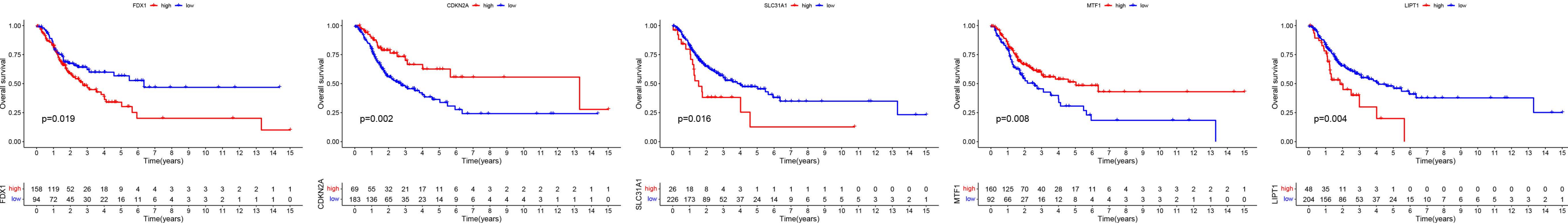

B

Jab1 Low

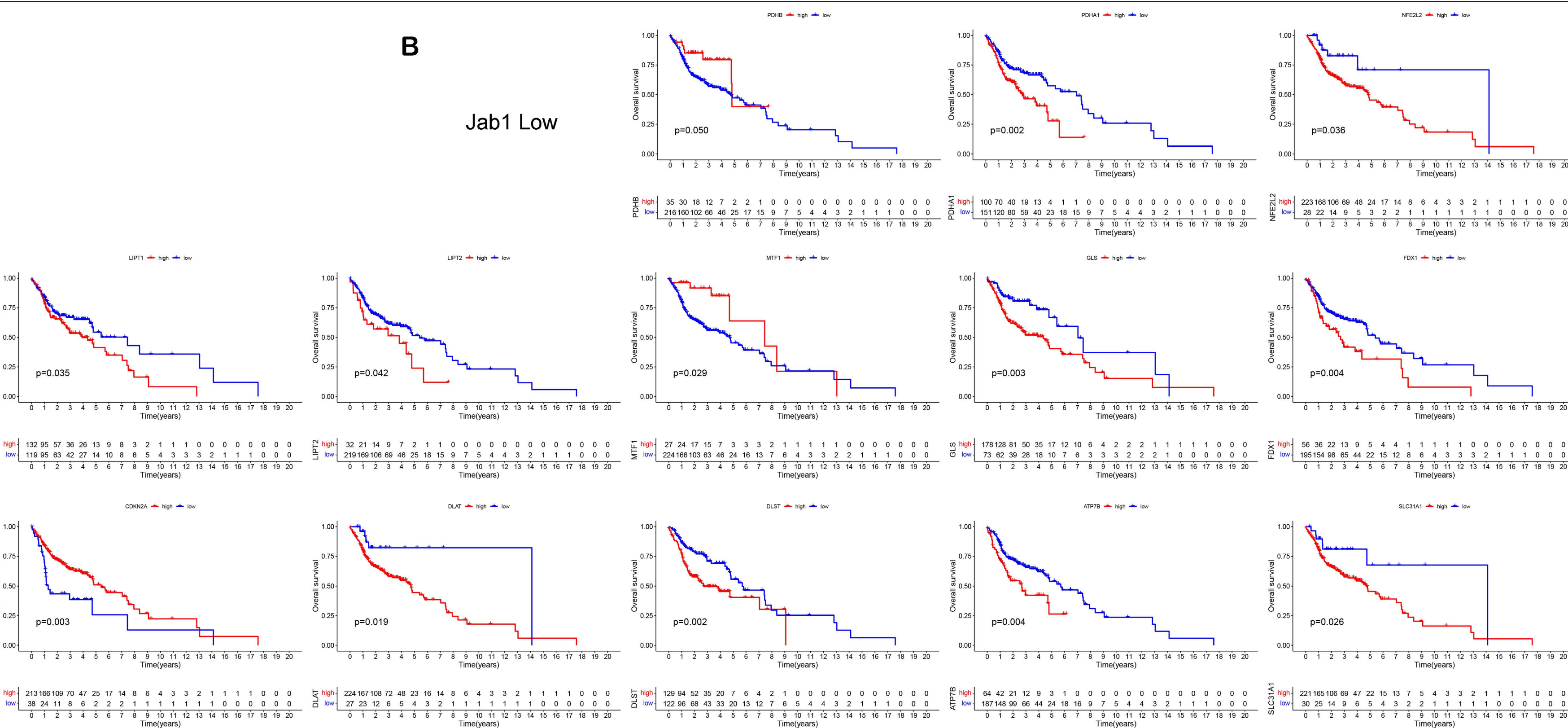

Supplement: Supplementary Figure 1 — Cuproptosis related genes for stratification in tumor cell-enriched region and immune cell-enriched region region. (A) NPC patient status distribution (PCA group) in tumor cell-enriched region and immune cell-enriched region. (B) Differential analysis in immune cell-enriched region, green for downregulation, red for upregulation, log|FC|>1 and P<0.05. (C) Univariate Cox analysis in immune cell-enriched region. (D) Principal component analysis in immune cell-enriched region. (E) Survival analysis of genes screened by univariate Cox regression analysis in tumor cell-enriched region and immune cell-enriched region. [file DataSheet_1.pdf]
